# A biostatistical support system in health sciences: is this sustainable in a resource-restricted environment?

**DOI:** 10.1186/s12961-019-0470-x

**Published:** 2019-07-05

**Authors:** Elena Libhaber, Tobias Chirwa, Beverley Kramer

**Affiliations:** 1 Health Sciences Research Office, Faculty of Health Sciences, University of the Witwatersrand, Johannesburg, South Africa; 2 School of Clinical Medicine, Faculty of Health Sciences, University of the Witwatersrand, Johannesburg, South Africa; 3 School of Public Health, Faculty of Health Sciences, University of the Witwatersrand, Johannesburg, South Africa; 4 School of Anatomical Sciences, Faculty of Health Sciences, Univerisity of the Witwatersrand, Johannesburg, South Africa

**Keywords:** Biostatistics, biostatistical support system, research training, financial sustainability

## Abstract

**Background:**

Training in biostatistics is important for strengthening capacity in health research. This is particularly true for Africa, where research output in the health sciences has been low. Training initiatives for the continent are therefore essential. The aim of the present study was to analyse the quality and financial sustainability of the expanded biostatistical support system at a South African health sciences institution between 2013 and 2017.

**Methods:**

A cross-sectional investigation of the initiatives created between the years 2013 and 2017 in the University of the Witwatersrand, Faculty of Health Sciences Research Office was undertaken. An assessment of the one-on-one consultations carried out by postgraduate students and staff, financial costs of the support system and the number of postgraduate student graduations were analysed.

**Results:**

The number of statistical consultations increased over the period examined. The consultations were highly recommended by the postgraduate students and staff (consulters). A clear rise in the number of Masters and PhD student graduates and an increase in research units were observed from 2013 to 2017, although these cannot be solely associated with the biostatistical support system. The finances for maintaining the support system are cost effective as the number of graduates increases. The total cost to the Research Office is US$ 225 per graduate per annum.

**Conclusions:**

The expansion of the biostatistical support system has indirectly contributed to an increased number of graduates and research publication units in the institution. While the current finances support the system, any increases in enrolments or growth in diversification of biostatistical requirements may place a strain on the financial sustainability. This service is of value to developed and developing countries.

## Introduction

The African continent has one of the highest burdens of disease, yet African research has been described as “*moribund*” [1] and lags behind the rest of the world with respect to scientific publishing [2]. There is also a limited amount of published research “*done in Africa for Africa*” [1]. Limited research capacity in Africa is an important factor that impedes the continent’s ability to produce health research [3–5]. For strategies on improving health outcomes to be effective, health sciences research and capacity development in this arena is critical. In South Africa, a middle-income country with a quadruple burden of disease [6], the strengthening of capacity development and of research skills is essential in order to support health research [7–10]. One such skill, fundamental for quality and relevant health research, is biostatistics.

Not only is biostatistics essential for clinical decision-making and the analysis of data for informing health policy [11] but also in the design, management and analysis of data in both applied and basic medical science research. Without knowledge of this key skill, it is not only difficult to plan and execute research but also to understand published research in the health sciences fields. Clinicians believe that an understanding of biostatistics would benefit their careers [12]. Knowledge of biostatistics may also result in more efficient and effective cost–benefit solutions to financially constrained health departments and assist with medical decision-making. Furthermore, the demand for skills in biostatistics to support research in clinical trials, genomics, proteomics and bioinformatics [13] predicts a need, not only to train more biostatisticians, but also to expand their areas of training. Thus, it appears that knowledge of basic biostatistics is fundamental for all medical doctors and biomedical researchers in current and future practice [14].

In South Africa, most students graduate from secondary schools with a huge knowledge deficit in mathematics and science [14–17]. Such students will thus require strong educational input in biostatistics at tertiary level in order for them to fulfil their roles as clinicians and biomedical scientists in the next millennium. This is supported by Chima et al. [14], who demonstrated poor baseline knowledge of biostatistics among biomedical researchers and postgraduate students in South Africa.

While a Master’s programme in epidemiology and biostatistics was already in existence in the Faculty of Health Sciences, University of the Witwatersrand [18], a biostatistical support system for all postgraduate students and staff was initiated and operationalised by the Health Sciences Research Office (HSRO) of the Faculty of Health Sciences (FHS), University of the Witwatersrand (Wits) in 2008. Between 2008 and 2012, the system consisted of modules in biostatistics at entry and advanced level to postgraduate students, a module aimed specifically for supervisors, and one-on-one consultations for students and staff [19, 20]. This system resulted in an increased number of graduates and publications, although we expressed caution in linking these outcomes directly to the system. The setting up of the initial system was carried out on a limited budget but would be affordable to institutions in a resource-restricted environment [19]. As the uptake of biostatistical and research methodology courses by staff and students increased from 2010 to 2012, so too did the number of graduates and publications, although other strategic initiatives may also have contributed to these increases [20]. An assessment of one-on-one consultations over the period 2008–2012 was also undertaken. While the initial biostatistical courses and consultations were in place, there was a need to expand and sustain the ongoing programmes. Expansion occurred in relation to the burgeoning needs of the Wits FHS from 2013 onwards. These needs were related to a perceived increase in postgraduate student registrations and entry of increased numbers of staff and students into research areas related to clinical trials, proteomics and mixed methods analysis. This perceived growth translated into an observed demand on the biostatistical support initiatives around the years 2011–2012.

Thus, the aim of the present study was to describe and analyse the nature and quality of the biostatistical initiatives that allowed expansion of the existing support system in the Wits FHS between 2013 and 2017. In addition, we wished to determine whether the support system is sustainable in terms of financial costs in a limited-resource developing country.

The objectives of this study were to (1) describe the initiatives that were added to the existing support system; (2) assess the quality of the biostatistical support system by the evaluation of the one-on-one consultations and to compare the number of graduates and publication units in relation to the foregoing assessment from 2008 to 2012; and (3) to determine the current financial budget of the support system.

## Methods

Ethics clearance was obtained from the Human Research Ethics Committee (Medical) of the University of the Witwatersrand (M1211112; W-CP-180280-2).

### Study site

The study was conducted in the Faculty of Health Sciences, University of the Witwatersrand, which is based in Johannesburg, Gauteng Province, South Africa. The Faculty not only trains undergraduate students in medicine and allied health sciences professions but also caters for postgraduate students in these fields. Such students are from within South Africa as well as across the African region. Most of the limited training in biostatistics occurs at postgraduate rather than undergraduate level [19].

### Study design

This descriptive, cross-sectional investigation examines the initiatives put in place in a biostatistical support system based in the Wits HSRO. It extends the assessment on the feedback on consultations of postgraduate students and staff on the support system [19].

The outcomes assessed were (1) the quality of the one-on-one consultations; (2) the number of graduates and publication units compared to those of 2008–2012; and (3) the financial sustainability of the current support system.

### Walk-in consultations

The existing platform for one-on-one walk-in biostatistics consultations with tutors increased from 9 h per week in 2012 to 16 h per week from 2014, due to increased demand by postgraduate students and staff. In a similar way to the 2009 survey [19], the current study utilised closed, anonymous questionnaires that were completed by individuals following walk-in one-on-one biostatistical consultations with a tutor. Questions in the brief assessment related to the quality of the consultation were rated on a Likert scale of 1–4, where 1 was ‘very bad’ and 4 was ‘excellent’. The questions related to learning from the consultation, confidence to analyse independently after consultation and whether the service would be recommended were answered on a scale of yes/no/not sure. The questionnaire was optional and anonymous, was undertaken at the end of a consultation and took no longer than 5 min to complete. The questionnaire was then placed in a sealed box.

### Additional workshops/modules

Additional biostatistics short courses, workshops and activities became necessary following initial set-up of support. An introductory course in qualitative methodology was established in 2012 due to increased demands from students and researchers for qualitative and mixed methods analysis. A workshop on data management and analysis, utilising the postgraduate student/staff own data, was initiated in 2014.

### Capacity development

The tutors performing biostatistical one-on-one consultations and facilitation are postgraduate students undertaking either a MSc or PhD degree in Biostatistics in the FHS. From 2018, these duties became part of their curriculum. The tutors are closely supervised by a senior biostatistician.

### Financial costs

The costs of senior biostatistics staff, tutors and facilitators were calculated from data received from the Wits HSRO. The average annual charges for licenses of biostatistical packages were calculated over the last 5 years.

The postgraduate students who acted as tutors in the one-on-one consultations that were ‘walk-in’ sessions (i.e. without booking) are not paid as such duties became part of their curriculum. If advanced statistical support was required, the tutors referred such cases to senior biostatisticians on a booking system.

Workshops and consultations are free to postgraduate students and staff within the Wits FHS. However, courses of over 4 hours in duration were charged at a nominal fee of R300 (~US$ 23) per course for students. This amount is paid to the Wits Faculty Research Office by departments.

### Number of graduates and research publications

The number of graduates and the publication units were used as an indirect proxy of achievement of the system. Units are the value of measurement used by the South African Department of Higher Education and Training for South African tertiary Institutions in relation to publication output. This measure is utilised as a comparator across institutions instead of publication numbers. One unit does not equate to one publication.

Data were extracted from the Institution’s database on postgraduate enrolments and graduations.

### Data management and analysis

Data was entered into an Excel spreadsheet and imported to STATA version 14 for analysis. The analysis of the data was descriptive and the results were presented in frequency tables with corresponding percentages.

## Results

### Additional initiatives 2013 to 2017

**Fig. 1:**
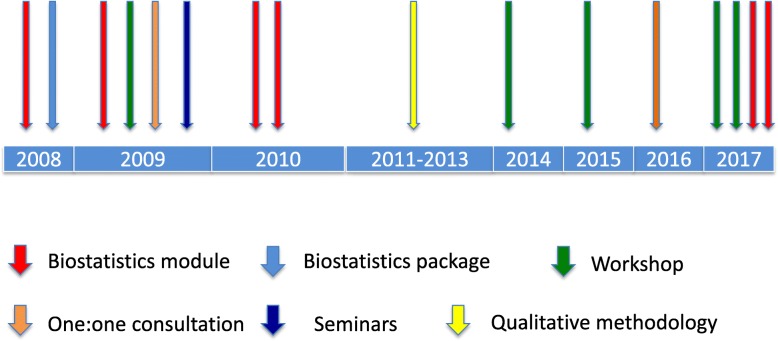
Parallel and sequential initiation (by year) of the biostatistics activities introduced by the Health Sciences Research Office for staff and postgraduate students of the Wits Faculty of Health Sciences

Additional initiatives specific to biostatistical education were introduced between 2013 and 2017 and are depicted in Fig. 1.

As part of the qualitative research methodology first introduced in 2012, workshops with the software packages NVivo (trademark) or MAXQDA (trademark) were initiated in 2017.

Several additional biostatistical modules were introduced to complement those already existing. These included practical-oriented workshops for postgraduate students analysing and utilising their own research data (2014); two applied hands-on descriptive and inferential biostatistics modules where students worked on data provided by the facilitator (2015) using statistical programmes (Statistica and STATA); one-on-one consultations for PhD students and staff members, particularly established researchers (2016) (Fig. 1); and a course on questionnaire design (2017). The workshops introduced in 2014 were aimed at small groups (maximum 12 students) and occurred monthly. Both data management and data analysis were performed during these sessions. In 2017, the data management and data analysis workshops were offered alternatively on a monthly basis.

Monthly biostatistician tutor meetings with senior biostatisticians were initiated in 2015 in order to periodically discuss any difficulties encountered or to offer guidance on alternative methods of consultation. Training of the tutors in the use of other biostatistical programmes (Statistica and STATA) contributed to the development of the skills of these individuals.

### Individuals attending, and evaluation of, one-on-one consultations 2013–2017

**Fig. 2:**
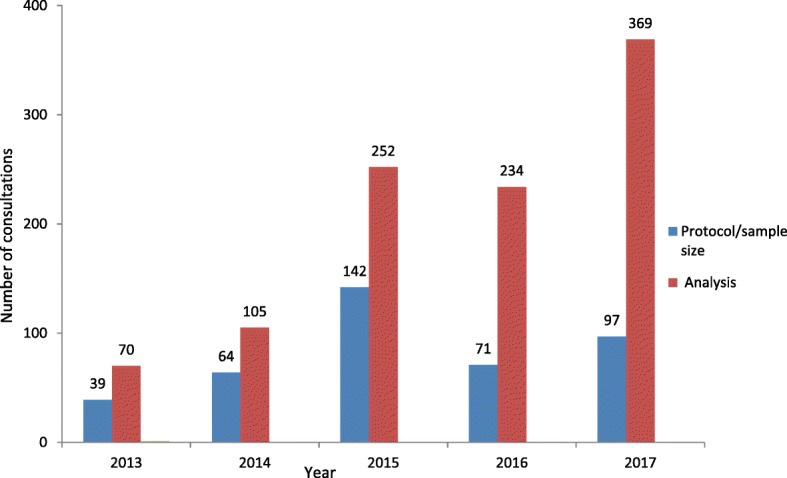
Number of consultations by nature of session held between 2013 and 2017

Table 1 shows the number of individuals who attended the consultations, while Fig. 2 shows the nature of the one-on-one consultations that occurred between 2013 and 2017. A total of 1077 evaluations were completed over the period 2013–2017 (Table 1). An increase in the number of one-on-one consultations occurred between 2013 and 2017 (Table 1). The majority of the consultations comprised queries on data analysis (between 70% and 89%). Consultations required by postgraduate students undertaking a Master’s degree constituted 37–62% of the total queries versus 11% to 18% for PhD consultations across the years.

**Table 1:** Number of individuals by degree type attending one-on-one consultations between 2013 and 2017

Year	Number of consultations	Masters by coursework *n* (%)	Masters by research *n* (%)	PhD *n* (%)	Research not for degree purposes *n* (%)
2013	99	40 (40)	20 (20)	18 (18)	12 (12)^a^
2014	137	51 (37)	26 (19)	24 (17.5)	2 (1.5)
2015	351	218 (62)	77 (22)	38 (11)	12 (3)
2016	283	132 (47)	45 (16)	36 (13)	17 (6)
2017	414	169 (41)	94 (23)	55 (13)	19 (4.5)

^a^As some data were not completed by the consultants, the percentages in the table do not always total 100%

Table 2 presents the results of the feedback by postgraduate students and staff members on the one-on-one consultations by year. Approximately 75% of consultees completed the questionnaire over the 5-year period. The appraisal of the consultations described as ‘excellent’ increased from 46% in 2013 to 65% in 2017. Over all the years surveyed, the majority (94–99%) of the consulters recommended the service provided.

**Table 2:** Appraisal by staff members and postgraduate students of the one-on-one consultation by year

Year	2013 (*n* = 37)	2014 (*n* = 117)	2015 (*n* = 311)	2016 (*n* = 247)	2017 (*n* = 365)
Appraisal of consultations					
Excellent	17(46)	69 (59)	166 (53)	152 (62)	237 (65)
Very good	16 (43)	34 (29)	105 (34)	60 (24)	84 (23)
Good	4 (11)	10 (8.5)	36 (12)	20 (8)	27 (7)
Bad and very bad	0	0	0	1 (0.4)	0
Learning from consultation					
Yes	31 (84)	111 (95)	278 (89)	201 (81)	327 (90)
No	0	1 (0.9)	7 (2.3)	4 (1.6)	4 (1)
Not sure	6 (6)	3 (2.6)	26 (8)	29 (12)	15 (4)
Confident to analyse after consultation					
Yes	35 (95)	108 (92)	275 (88)	200 (81)	322 (88)
No	0	2 (1.7)	7 (2.3)	3 (1.2)	8 (2)
Not sure	2 (5)	2 (1.7)	26 (8)	24 (10)	18 (5)
Recommend the service to someone else					
Yes	36 (95)	110 (94)	310 (99.7)	231 (94)	343 (94)
No	0	0	1 (0.3)	1 (0.4)	1 (0.3)
Not sure	0	0	4 (1.3)	1 (0.4)	5 (1.4)

### Number of graduates and research units by publications

**Fig. 3:**
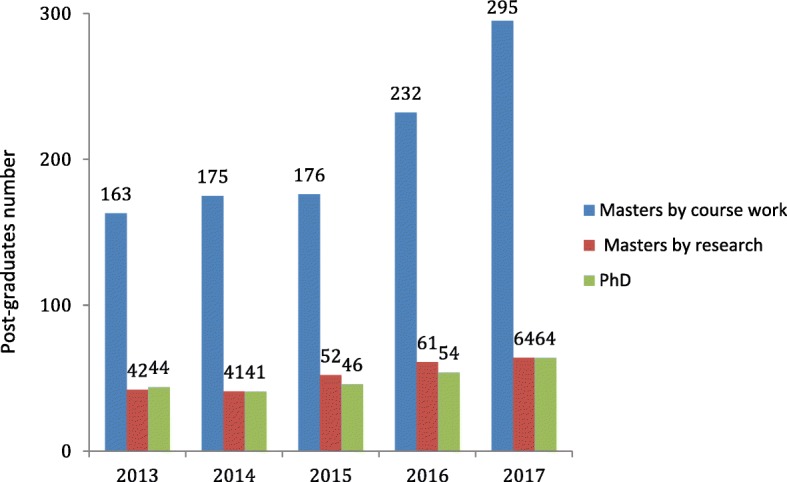
Number of graduations per degree type (2013–2017)

There was an average increase of 1.3% in enrolments of all postgraduate students between 2013 and 2017. During the same time period, an average of 17% of students undergoing a Master’s degrees by coursework (MScC), 10.5% undergoing a Master’s by research and 10% undergoing a PhD graduated (Fig. 3).

A clear rise in the average number of PhD and MScC graduates was observed from 2013 (PhD, *n* = 44; MScC, *n* = 163) to 2017 (PhD, *n* = 64; MScC, *n* = 295). There was also a slight increase in graduations for students undertaking the Master’s degree by pure research, from 42 to 64 over the same period of time (Fig. 3).

A marked increase in the number of research units by publication was achieved in the FHS in 2017 (*n* = 585.35) compared to 2012 (*n* = 291.65).

### Financial aspects (2017)

#### Overall financial cost of operating the biostatistical support service

A biostatistician based in the HSRO costs approximately US$ 57,692 per annum. The tutors (*n* = 4) who consulted one-on-one with postgraduate students and staff were together paid US$ 538 per month for 11 months (US$ 5918 in total per annum), while facilitators (*n* = 2) for workshops and courses were each paid US$ 77 per month for 11 months, which totalled US$ 1694 per annum.

Licenses for the two statistical packages, STATA and Statistica, cost the HSRO ~US$ 15,385 in 2017. The qualitative software licenses for NVivo and MAXQDA together cost US$ 6154. While the HSRO did not pay for the use of infrastructure nor the use of the computer laboratories, additional costs incurred were US$ 8408 as payment for a part- time (50%) administrator for the courses. Thus, a total of approximately US$ 95,251 per annum is required to sustain the biostatistical training. When considering the number of graduates in 2017 alone (*n* = 423), biostatistical support was provided at a cost of US$ 225 per graduate.

#### Income generated through the provision of biostatistical support services

The HSRO charges a nominal fee for courses of ~ US$ 23 per course for postgraduate students. There is no charge for staff attending these courses. In addition, individuals external to the University are charged ~US$ 192 per course. A total of ~US$ 4154 from students, ~ US$ 1923 from external individuals and ~US$ 673 from the issuing of software programme discs resulted in an income of ~US$ 6965 by the HSRO from these courses in 2017. The latter contributed to the costs of the facilitators, software packages and stationery used in the courses/workshops.

## Discussion

The expansion of the modules of the biostatistics support system augmented the need for increasing student numbers and affected the nature of the research being undertaken. The quality of the one-on-one consultations remained high during the 5-year period reviewed. Concomitant with the increased numbers of consultations was an increase in the number of graduates and research units over the same period. Financial costs of the support system during this period were cost-effective.

One of the reasons for increasing the resources for biostatistics training in our institution was the introduction of a requirement by the Health Professions Council of South Africa [21], in 2010, for clinicians who were specialising in specific disciplines to undertake a research project as part of their Master’s degree, which became mandatory in 2011. Hence, the increase in the number of graduates may also be as a result of this implementation as these students required statistical support for project completion.

There are few case studies on the investment in biostatistical skills in health sciences to build research capacity in the developing world. This study sought to evaluate the quality and financial sustainability related to the additional activities instituted in biostatistical support since 2013 in the Wits FHS. Ideally, one would like to see a cohort effect of individuals who have gone through such support. It is difficult to find an objective mechanism for measuring success in relation to implementation of strategies [5]. However, there has been a growth in high impact factor publications (IF ≥20) from Wits FHS. In the period 2000–2012, there were 28 high impact factor publications, increasing to 39 over the period 2013–2016 (Health Sciences Research Office annual reports 2008–2016, Internal publication). It should be noted that this increase might not be directly linked to the Biostatistics support system, as the target of the support system is postgraduate students and more junior researchers. In addition, accompanying the increase of almost 10% in registrations over all the degree types between the periods 2010–2012 and 2013–2017 in the Faculty, the proportion of graduates increased by approximately 30% over the same period of time.

Notwithstanding these limitations, we note that the number of graduates and research publications lead to great benefit for the Institution as recognition in the South African context. South African Institutions keenly follow the international Institutional Rankings and are highly competitive both locally and internationally in achieving higher rankings. In 2017, Wits was ranked 101–125 out of 500 institutions in the clinical, pre-clinical and health category of the Times Higher Education World University Rankings. Thus, although we cannot link increases in publications and number of graduates directly to the biostatistics strategies introduced at Wits as other factors may be involved, it is, however, a partial indicator of the value of the training in biostatistics. Our postgraduate programmes have a structured coursework and research component. Coursework is completed on time but the challenge has been completion of the research, since most students lack statistical guidance for analysis. This biostatistics support system has bridged that gap and led to an increase in completion of research reports and, hence, graduations. The original biostatistical courses were initiated in 2008 and one-on-one consultations initiated in 2009. Hence, the number of graduating students in relation to the biostatistical support system were reported from 2010 [19]. Compared to the previous period [19], the numbers of graduating students in all degrees has increased. This can be indirectly associated with the increased biostatistical initiatives in recent time periods.

The Wits FHS has also seen increasing numbers of publications emanating from students work. More students have been accessing the biostatistical support system for consultations on statistical analysis of their research. The students are keen to publish their findings and this support system provides one mechanism for directing reports into publications.

On analysis of the survey on one-on-one consultations, a similar percentage of students (95.34%) used the service over the years 2013–2017 to the number of students that Chirwa et al. [19] recommend the service to between 2010 and 2012 (95.8%). The continued excellence of the consultations has borne fruit considering the increased throughput of postgraduate students. As these consultations are provided to individuals from a variety of disciplines by a central group of biostatisticians and biostatisticians-in-training, the model is similar to that of the “Hub-and-Spoke Model” described by Earnest [22]. However, in the case of consultations at Wits, these do not generally result in collaborations in which the biostatistician is included as an author on an article [22].

Development of a Masters’ programme in the field of biostatistics within the health sciences is not only important for upskilling those clinicians and researchers who will undertake research in the expanding fields of genomics, proteomics, metagenomics and clinical trials [23], but is also of importance in providing a pipeline of skilled biostatistics tutors [13, 23]. This has also proven to be a cost saving measure as the curriculum for the Master’s programme includes training on consultation. In this regard, the Wits FHS no longer pays the tutors for their services. Of value is that some biostatisticians are absorbed into research entities thus increasing the pool supporting analysis at the Wits FHS.

Related to postgraduate courses is the need to initiate training in biostatistics at an earlier stage of allied health sciences undergraduate programmes. More advocacy is needed for the insertion of biostatistics into undergraduate programmes in order to build on basic skills at the postgraduate level [14]. Basic biostatistics courses have been initiated in research methodology courses offered at undergraduate level in the Faculty in order to build graduates with competent skills in biostatistics and also as a pipeline of postgraduate students in health sciences fields. In fact, Mostert [15] argues that biostatistics, epidemiology and evidence-based medicine should be taught as a continuum in the medical curriculum.

The demand for biostatistics, biostatistics training and biostatisticians exceeds the current capabilities of health sciences facilities [13], particularly as biostatistics plays a large role in ‘big data’ research. Demand for biostatisticians far exceeds the supply in the United States [13], and in Africa there is a marked need for growth in this sector. Kellerman et al. [18] showed that investing in research training in African institutions resulted in the retention of graduates in Africa. Biostatistics has an unparalleled opportunity to contribute to Africa’s health needs as a fundamental of health research.

In relation to keeping costs minimal in a resource-restricted environment, the use of non-paid tutors for consultations seems a cost-effective alternative in providing a critical mass of assistance and mentors in the Faculty. In addition to spreading the financial burden, a recommendation is that this could be run as an integrated programme within existing entities with such expertise. In our case, the pooling of biostatistics resources between the Wits School of Public Health and HSRO has been cost-effective in driving activities for statistical support. The Wits FHS only pays for one biostatistician because of the shared roles at its disposal. The cost to graduation of US$ 227 per postgraduate student is cost effective. Of course, following training, Master’s and PhD graduates with these skills are engaged by commerce, where the enticement of a larger salary than in academia plays an important role.

When compared to the Wits FHS biostatistical initiative in 2012 [19], the current system has increased in cost (2012, US$ 68,000; 2017, US$ 96,154). However, the cost per graduate in 2017 (US$ 227) decreased as the number of graduates increased. Hence, the support system is viable in the current economic climate that exists in the institution. However, further increases (e.g. student numbers or expansion of research areas) will place a burden on the finances of the system.

Offering the courses to external participants has financial benefits and such participants should be targeted more in order to increase funding to support the system going forward. Only 10% of such participants attended the course but contributed a third of the funds raised through short courses.

While biostatistics and epidemiology are of great importance in producing research and developing capacity, institutions in South Africa do not mention this skill when developing research strategies [24, 25].

Chima et al. [14] showed an improvement in knowledge of biomedical researchers and postgraduate students following the attendance of a short biostatistics course. While recognising the importance of courses, we have attempted to go one step further in providing ongoing support to postgraduate students and staff in addition to short courses by means of consultations and workshops. Solid biostatistical support systems, particularly with the advent of ‘big data’ analysis, are becoming challenging. In order to keep Africa from lagging in health sciences research with respect to the rest of the world, investing in the maintenance and growth of biostatistical support systems is of great importance. The model has become more cost effective as more students use and graduate in the system. This model, created by the Wits Health Sciences Faculty for biostatistics support, may assist other health sciences institutes in developing countries with instituting training at low cost.

### Limitations of study

This is a cross-sectional study and thus there was no intervention, and hence no follow-up. There is no control group, as we do not have data for those students not attending the Biostatistical support unit. While Wits is an institution in a middle-income country, similar data from other institutions in the country are not available for comparison. The number of one-on-one consultations is based on the number of completed evaluations.

## Conclusion

The financial sustainability of the Wits FHS biostatistics support system in an ongoing restricted economic environment was of concern but has proven to be cost effective as more graduations occur. The support system may be a model to other institutions in similar low- and middle-income settings, and appears to have contributed to increased postgraduate throughputs, particularly PhDs. Challenges that need to be addressed include continued funding at the current or increased level and increasing the pool of skilled biostatisticians who can be involved in peer teaching. Thus, the biostatistical service is worthy of continued support in the Faculty. Over the long term we envisage sustainability of the support system as critical for research.

## Data Availability

Data is available from the Health Sciences Research Office of this institution. Additionally, data are available from the authors upon request and with permission of the Health Sciences Research Office.
